# Supplementary material for: Self-Regulation of Attention in Children in a Virtual Classroom Environment: A Feasibility Study
Source: Bioengineering (Basel). 2023 Nov 24;10(12):1352. doi: 10.3390/bioengineering10121352 (PMC10741222; doi:10.3390/bioengineering10121352)
Supplement: Supplementary file 1 [file bioengineering-10-01352-s001.zip › bioengineering-2710438-supplementary.pdf]

Self-Regulation of Attention in Children in a Virtual Classroom Environment: A Feasibility Study

Supplementary Materials:

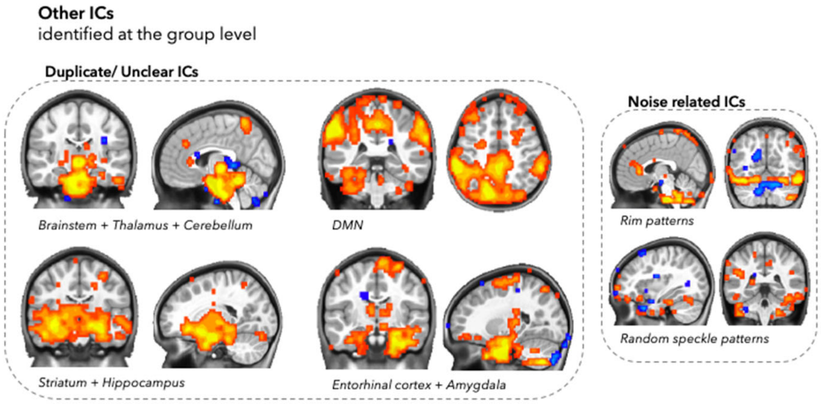

Figure S1. Example of other ICs.

Table S1. Cluster table.

Cluster size	x	y	z	Regions
623	9.5	-83.5	55	R Precuneus
310	-8.5	45.5	13.8	L Superior Frontal Gyrus
234	-68.5	-53.5	6.2	L Middle Temporal Gyrus
153	-50.5	30.5	-8.8	L Inferior Frontal Gyrus
101	69.5	-32.5	32.5	R SupraMarginal Gyrus
92	60.5	27.5	2.5	R Inferior Frontal Gyrus
64	9.5	-86.5	2.5	R Calcarine Gyrus
53	54.5	27.5	36.2	R Middle Frontal Gyrus
